# Editorial for the Special Issue “Molecular Mechanisms and Regulation in Allergy and Immune Diseases, Immunodeficiencies, 2nd Edition”

**DOI:** 10.3390/cimb48090885

**Published:** 2026-08-31

**Authors:** Kinga Lis

**Affiliations:** Department of Allergology, Clinical Immunology and Internal Diseases, Ludwik Rydygier Collegium Medicum in Bydgoszcz, Nicolaus Copernicus University in Torun, ul. K. Ujejskiego 75, 85-168 Bydgoszcz, Poland; kinga.lis@cm.umk.pl

Allergies and congenital and acquired immunodeficiencies represent a significant problem in the modern world, one that is constantly growing in scale. Continuous research elucidating the mechanisms of these diseases allows for a better understanding of their mechanisms, including at the molecular level, providing a basis for the development of new diagnostic and therapeutic tools [1,2,3,4].

Due to the importance of these issues, following the publication of the first Special Issue on “Molecular Mechanisms and Regulation in Allergy and Immune Diseases, Immunodeficiencies, 2nd Edition” we have decided to continue our discussion of this topic in this next, second edition. The articles included in this Special Issue explore the mechanisms of immune dysfunction, including allergies, the properties and stability of allergens, and the potential practical applications of this knowledge in the diagnostic and therapeutic processes of these disorders.

In this second edition, we have focused on expanding our knowledge of sensitizing proteins, their structure, and the relationship between possible modifications and changes in their stability [5]. This is particularly important in the context of unstable allergens, which are destroyed during the production or storage of diagnostic reagents and vaccines. We have also noted rare hidden allergens [6,7] and have emphasized that substances perceived as harmless or health-promoting can also cause allergies or toxic effects.

We have noticed that it is worth subjecting ancient, natural, or traditional medicine to contemporary analyses [8]. In line with this trend, the articles in this Special Issue discuss current biological therapies for asthma, atopic dermatitis, and allergies [contribution 1], along with the search for new strategies for the prevention and treatment of allergic diseases using preparations derived from traditional natural medicine or physical factors [contribution 2]. We have also noted that understanding the molecular basis of various tissue and organ dysfunctions [9] and their interrelationships at the molecular level [10] can help to expand the spectrum of diagnostic tools and identify new, important therapeutic targets.

The *CIMB* editorial office considered a total of 13 manuscripts for inclusion in this Special Issue. All were thoroughly reviewed by the Scientific Editor(s) and Reviewers, who are specialists in allergology and clinical or laboratory immunology. Ultimately, eight articles have been included, comprising four original articles and four review articles. Although the second edition of “Molecular Mechanisms and Regulation in Allergy, Immunological Diseases, and Immunodeficiencies” received a somewhat smaller response than the previous one, we hope that its goals have been achieved. This would not have been possible without the contributions of all the esteemed authors who have chosen our Special Issue for the publication of their research or reviews. It would also not have been possible without the participation of the Reviewers, who invested their time into thoroughly and compassionately evaluating the submitted manuscripts, and all the members of the Editorial Board who actively participated in the editorial work on this issue. I would like to sincerely thank everyone involved.

We would also like to announce that we are planning to continue our exploration of the themes of this Special Issue in a future third edition. We invite authors to submit their manuscripts (original articles and reviews) for the Special Issue “Molecular Mechanisms and Regulation in Allergy, Immunological Diseases, and Immunodeficiencies, 3rd edition” as soon as it is launched.

## Data Availability

No new data were created or analyzed in this study. Data sharing is not applicable to this article.
